# Supplementary material for: An implementation science framework to understand low coverage in mass dog rabies vaccination
Source: PLoS Negl Trop Dis. 2025 Jul 23;19(7):e0012869. doi: 10.1371/journal.pntd.0012869 (PMC12342281; doi:10.1371/journal.pntd.0012869)
Supplement: S1 Checklist — (DOCX) [file pntd.0012869.s001.docx]

**Consolidated criteria for reporting qualitative studies (COREQ): 32-item checklist**

| **No. Item** | **Guide questions/description** | **Reported on Page #** |
| --- | --- | --- |
| **Domain 1: Research team and reﬂexivity** |  |  |
| *Personal Characteristics* |  |  |
| 1. Interviewer/facilitator | Which author/s conducted the interview or focus group? | VPS, RCN, EWD -page 6 |
| 2. Credentials | What were the researcher’s credentials? E.g. PhD, MD | PhD, PhD, DVM –page 6, 7 and 10 |
| 3. Occupation | What was their occupation at the time of the study? | Principal Investigator, Principal Investigator, Researcher – page 7 |
| 4. Gender | Was the researcher male or female? | Female, male, male – page 7 |
| 5. Experience and training | What experience or training did the researcher have? | VPS has extensive experience in facilitating and conducting interviews, which is an integral part of her professional role. She has received formal training in qualitative research methods, including facilitation techniques, interview protocols, and data analysis. RCN has been working and training with VPS and conducting focus groups since 2016, joining as a co-facilitator, facilitator and as the principal interviewer for several years. EWD has been working for more than four years as a co-facilitator under the supervision of VPS – page 7 |
| *Relationship with participants* |  |  |
| 6. Relationship established | Was a relationship established prior to study commencement? | **Community participants.** “All participants had to be dog owners and had no prior relationship with the research team.” On page 8  **Health authorities and vaccine campaign implementers.** **“**Our research team has been working with health authorities in this region for about two decades – first, for studies related to Chagas disease, and since 2015, when rabies outbreak started, with authorities. Our study team did not have a prior relationship with the vaccinators who are volunteers.” – on page 8. |
| 7. Participant knowledge of the interviewer | What did the participants know about the researcher? e.g. personal goals, reasons for doing the research | Participants were informed about the researchers’ roles as facilitator and co-facilitators during the focus group. They were also made aware of the study's objectives, including understanding the barriers to mass dog rabies vaccination (MDVCs) during the written informed consent process. However, participants were not provided with detailed personal information about the researchers’ backgrounds or personal goals beyond the context of the study.  MDVC authorities and implementers know that the researchers are studying ways to reduce rabies transmission, including ways to optimize vaccination campaigns. They participated in a workshop with the research team in which they signed a one-time consent form that explained our team would be interacting and observing vaccination campaigns as part of our research, working collaboratively with the health authorities. Observations in the field were public.  “Before each focus group, the facilitator made aware of the study's objectives, including understanding the barriers to mass dog rabies vaccination (MDVCs) and reviewed the consent form with participants and obtained written consent regarding voluntary participation and agreeing to audio recording” – page 10. |
| 8. Interviewer characteristics | What characteristics were reported about the interviewer/facilitator? e.g. Bias, assumptions, reasons and interests in the research topic | The facilitator and co-facilitators were trained researchers with extensive experience in qualitative methods and public health, specifically in the context of zoonotic diseases. While the researchers have a professional interest in rabies control and vaccination strategies, every effort was made to minimize bias by adhering to a focus group guide and ensuring that participants felt free to share their views without influence. The researchers’ interest in the study topic was motivated by a desire to understand the barriers to vaccination and improve public health outcomes in endemic areas.  “We also emphasized at the start of each focus group the importance of obtaining different points of view and that there were no correct or incorrect answers to ensure that participants felt free to share their views without others’ influence.” – page 10 |

| **Domain 2: study design** |  |  |
| --- | --- | --- |
| *Theoretical framework* |  |  |
| 9. Methodological orientation and Theory | What methodological orientation was stated to underpin the study? e.g. grounded theory, discourse analysis, ethnography, phenomenology, content analysis | The study was underpinned by a qualitative methodological approach, specifically using focus groups to explore participants' perspectives. We also conducted a workshop for MDVC implementers and authorities. The Consolidated Framework for Implementation Research (CFIR) was applied as the theoretical framework to identify barriers and facilitators in the implementation of MDVCs. – page 11 |
| *Participant selection* |  |  |
| 10. Sampling | How were participants selected? e.g. purposive, convenience, consecutive, snowball | **Community Participants.**  “Our research team used purposive sampling to recruit 56 community members for focus groups through a two-phase process, selecting women who owned dogs from four urban and three periurban areas from a randomly selected area of about nine neighborhood blocks that were at least 5 blocks away from a health facility to minimize potential bias from living near a health facility and being more exposed to health-related information.”  **MDVC authorities and implementers.** We organized a daylong workshop in Arequipa for MDVC implementers and authorities. We randomized implementers into different groups to ensure diversity and enhance discussion.  “We organized an in-person daylong workshop in Arequipa, inviting 80 MDVC implementers and authorities. During this workshop, attended by 69 participants, we conducted 14 focus groups with implementers: 13 with four implementers each and one with six implementers. These participants represented 20 different *microreds*. We randomized implementers into each group to ensure diversity and enhance discussion. Additionally, we held a focus group with the 11 authorities attending the workshop.” - Page 9 |
| 11. Method of approach | How were participants approached? e.g. face-to-face, telephone, mail, email | **Community Participants.** Participants were approached face-to-face for recruitment. Community members were recruited through door-to-door visits conducted two hours before the focus groups. “We conducted door-to-door (face-to-face) recruitment two hours before the event, enlisting 25 participants from periurban and 31 from urban areas.”  **MDVC authorities and implementers.** For the workshop, participants were approached in person before the event. “We organized an in-person daylong workshop in Arequipa, inviting 80 MDVC implementers and authorities” – page 9 |
| 12. Sample size | How many participants were in the study? | A total of 125 participants were involved in the study. **Community Participants.** 56 community members (31 from urban areas and 25 from periurban areas).  **MDVC authorities and implementers.** 69 MDVC implementers and authorities. – page 9 |
| 13. Non-participation | How many people refused to participate or dropped out? Reasons? | 11 MDVC implementers and authorities received the personal invitation to participate in the workshop but they did not show due to their job responsabilities. – page 9 |
| *Setting* |  |  |
| 14. Setting of data collection | Where was the data collected? e.g. home, clinic, workplace | **Community Participants.** The data was collected in health centers, courtyards, and schools in Arequipa City, Peru.  “Focus groups were conducted in schools or courtyards” – page 10  **MDVC authorities and implementers.** The workshop was held during a daylong event in Arequipa. – page 9 |
| 15. Presence of non-participants | Was anyone else present besides the participants and researchers? | No, there were no non-participants present during the data collection. The focus groups and workshop included only the participants and the research team.  “Focus groups were conducted in schools or courtyards, without the presence of onlookers or non-participants”– page 10  The workshop began with a short sharing of preliminary findings of MDVC coverage in the different regions (anonymized to prevent judgement) and then proceeded to break out focus groups where data was collected. Only invitees were present. - Page 10 and 11 |
| 16. Description of sample | What are the important characteristics of the sample? e.g. demographic data, date | **Community Participants.** All women who owned dogs but had not participated in the last MDVC. Participants were recruited from both urban (31) and periurban (25) areas, with ages ranging from 18 to 76, and a median age of 40. Focus groups were conducted in 2022. – page 9.  **MDVC authorities and implementers.** “These participants represented 20 different *microreds*.” Workshop was conducted in 2023. Page 9. |
| *Data collection* |  |  |
| 17. Interview guide | Were questions, prompts, guides provided by the authors? Was it pilot tested? | Yes, the research team developed a focus group guide to explore factors influencing MDVC participation. The process involved comprehensive preparation, including expert facilitators.  “The research team developed a focus group guide to ensure similar questions were asked of each focus group to explore the main topic of interest: the factors at individual, community, and systems levels that contributed to the decrease in MDVC coverage post COVID-19.” - page 10 |
| 18. Repeat interviews | Were repeat interviews carried out? If yes, how many? | “No repeat interviews were conducted.” The focus groups were the primary method for data collection. - page 11 |
| 19. Audio/visual recording | Did the research use audio or visual recording to collect the data? | Yes, audio recording was used to collect data during all focus groups and workshops with participants, after obtaining consent.  “The facilitators informed the participants about the study’s objectives and obtained written consent for participation and audio recording” -page 10 |
| 20. Field notes | Were ﬁeld notes made during and/or after the interview or focus group? | Yes, field notes were made during and after the focus groups. Additionally, the research team took detailed field notes during MDVCs in 2022.  “For the MDVC observations, our rabies research manager (EWD) observed 41 MDVCs in action, from June to October 2022. He recorded notes and observations on a google document that was shared between him and the project PI (RCN) and discussed with the team at weekly meetings.” – page 11. |
| 21. Duration | What was the duration of the interviews or focus group? | “The community focus groups lasted around 60-90 minutes.” – page 10.  The workshop with MDVC implementers and authorities lasted a full day.  We organized an in-person daylong workshop in Arequipa, inviting 80 MDVC implementers and authorities”– page 9. |
| 22. Data saturation | Was data saturation discussed? | Yes, data saturation was discussed. “After achieving theme saturation in five focus groups, we conducted two additional groups to confirm no new topics emerged.” – page 9. |
| 23. Transcripts returned | Were transcripts returned to participants for comment and/or correction? | No, the transcripts were not returned to participants for comment or correction. “All data was analyzed without returning transcripts to participants (whether community or MDVC implementers)”. – page 11 |
| **Domain 3: analysis and ﬁndings** |  |  |
| *Data analysis* |  |  |
| 24. Number of data coders | How many data coders coded the data? | The data was coded by two coders. “The research team (LOC, JC) analyzed the double-transcripts using Dedoose version 9.0.90 for all focus group, workshop, and field note data” – page 11 |
| 25. Description of the coding tree | Did authors provide a description of the coding tree? | Yes, the authors provided a description of the coding process. “Once they conducted the last focus group, the team met to discuss the overall themes that had emerged inductively in preparation for the development of the codebook. After finalizing the workshop with MDVC implementers and authorities, the research team discussed new themes to add to the same codebook. The research team used a mix of inductive and deductive codes: CFIR domains and subdomains were used to organize data for analysis (deductive), using themes that emerged during data collection (inductive).” – page 11 |
| 26. Derivation of themes | Were themes identiﬁed in advance or derived from the data? | Themes were derived from the data. The research team conducted deductive analyses, identifying themes based on focus group discussions and field notes. – page 11 |
| 27. Software | What software, if applicable, was used to manage the data? | The software used for managing the data was Dedoose version 9.0.90. - page 11 |
| 28. Participant checking | Did participants provide feedback on the ﬁndings? | No, participants did not provide feedback on the findings after data analysis.  “All data was analyzed without returning transcripts to participants” – page 11 |
| *Reporting* |  |  |
| 29. Quotations presented | Were participant quotations presented to illustrate the themes/ﬁndings? Was each quotation identiﬁed? e.g. participant number | Yes, participant quotations were presented to illustrate the themes. Each quotation was explicitly identified by type and participant number. - pages 12-23.  E.g. "They used to put up posters, then they went from street to street during the whole week announcing when the campaign was going to take place" (Urban Community Member)” – pag 13 |
| 30. Data and ﬁndings consistent | Was there consistency between the data presented and the ﬁndings? | Yes, there was consistency between the data presented and the findings, as is presented in the discussion and conclusion sections. - pages 24-28 |
| 31. Clarity of major themes | Were major themes clearly presented in the ﬁndings? | Yes, most barriers appeared in the Implementation Process domain, followed by the Inner, Individuals, and Outer Setting domains – pages 11-22 |
| 32. Clarity of minor themes | Is there a description of diverse cases or discussion of minor themes? | Yes, we found them in the Innovation section  . – page 22 and 23. |
